# Postural Screening for Adolescent Idiopathic Scoliosis with Infrared Thermography

**DOI:** 10.1038/s41598-017-14556-w

**Published:** 2017-10-31

**Authors:** Garcia Kwok, Joanne Yip, Kit-Lun Yick, Mei-Chun Cheung, Chi-Yung Tse, Sun-Pui Ng, Ameersing Luximon

**Affiliations:** 1 Institute of Textiles and Clothing, The Hong Kong Polytechnic University, Hong Kong, China; 2 Department of Social Work, The Chinese University of Hong Kong, Hong Kong, China; 3 Centre for Orthopaedic Surgery, Central, Hong Kong, China; 4 Division of Science & Technology, Hong Kong Community College, Hong Kong, China

## Abstract

Adolescent idiopathic scoliosis (AIS) is a multifactorial, three-dimensional deformity of the spine and trunk. School scoliosis screening (SSS) is recommended by researchers as a means of early detection of AIS to prevent its progression in school-aged children. The traditional screening technique for AIS is the forward bending test because it is simple, non-invasive and inexpensive. Other tests, such as the use of Moiré topography, have reduced the high false referral rates. The use of infrared (IR) thermography for screening purposes based on the findings of previous studies on the asymmetrical paraspinal muscle activity of scoliotic patients compared with non-scoliotic subjects was explored in this study. IR thermography is performed with an IR camera to determine the temperature differences in paraspinal muscle activity. A statistical analysis showed that scoliotic subjects demonstrate a statistically significant difference between the left and right sides of the regions of interest. This difference could be due to the higher IR emission of the convex side of the observed area, thereby creating a higher temperature distribution. The findings of this study suggest the feasibility of incorporating IR thermography as part of SSS. However, future studies could also consider a larger sample of both non-scoliotic and scoliotic subjects to further validate the findings.

## Introduction

Adolescent idiopathic scoliosis (AIS) is a multifactorial, three-dimensional (3D) deformity of the spine and trunk. AIS can appear and sometimes progress during any of the rapid periods of growth in children^1^. Since the 1960s, research has shown that the early detection of scoliosis is effective in preventing its progression^2^; therefore, school scoliosis screening is recommended by researchers^3,4^. Early detection reduces the number of cases that require surgery through non-surgical interventions^5^. Despite criticisms of the effectiveness of school screening, primarily regarding unnecessary referrals and the high cost^6^, there are studies that justify the cost and effectiveness of school screening. For example, Lee *et al*.^7^ conducted a study on the cost of scoliosis screening in schools and concluded that the savings achieved by avoiding surgery compensate for the cost. Most of the cost lies in human resources and follow-up. Therefore, school scoliosis screening (SSS) should be conducted for the purpose of early detection. Fong *et al*.^8^ statistically examined the effectiveness of SSS by reviewing 36 studies. After conducting a meta-analysis, they could not provide a definite conclusion regarding the effectiveness of screening due to variations in screening methods and sample size. However, they found that the forward bending test (FBT) has a higher referral rate and less precision when it is the only means of assessment compared with that of other screening methods that use more than one form of screening. Based on this result, they suggested that SSS programmes that use the FBT as the only assessment tool are subjective and insufficient for providing a reliable and precise diagnosis; therefore, an additional test aside from the FBT is recommended.

Adams^9^, the leading expert on spinal deformity during his time, described the FBT as a screening technique to assess the likelihood of scoliosis during a series of twelve lectures that were presented from 1860 to 1861^10^. The FBT is still widely used today because it is the most conventional means of screening for AIS, and the technique is simple, non-invasive and inexpensive. Despite these advantages, there have been numerous criticisms against the FBT, and many researchers have reached similar conclusions to that reported by Luk *et al*.^3^, namely, the technique results in high false-positive and false-negative rates^5^, resulting in misdiagnoses and unnecessary referrals. Alternatives to the FBT include optical methods, such as Moiré topography, which has been used to obtain contour photographs of the shape of the back of scoliosis patients to assess the degree of deformity^11^. In Hong Kong, both the FBT and Moiré topography have been applied in school screening programmes. The former test is used to screen out most of the non-scoliotic cases. Children with an angle of trunk rotation (ATR) that is over 5 degrees are referred to a special assessment centre to be further examined using Moiré topography before they are taken to a specialist hospital for further diagnosis. When these two tests constitute the most important components of a scoliosis screening programme, the programme is predictive and sensitive, with a low referral rate^12^. However, one of the more recent techniques that is gaining popularity for measuring the surface topography of scoliotic patients is a light-sectioning method called rasterstereography (RS)^13,14^. This method not only has a certain level of accuracy, is inexpensive and does not require radiation exposure, but it is also quick^15^ and therefore may replace Moiré topography, which still results in some degree of ambiguity^16^, for routine clinical use in the near future.

Apart from the FBT, Moiré topography and RS, a commercial version of the Integrated Shape Imaging System (ISIS) is also a contender for scoliosis screening. It uses a structured light to project parallel fringes onto the back of a patient while a digital photograph is taken. The result is a colour 3D contour plot of the patient’s back in the coronal, transverse, and sagittal planes. Unfortunately, the use of surface topography as a screening tool still faces a number of limitations. For example, it requires specialized operational and evaluation skills. This tool is also not widely available^17^. Another major disadvantage is the length of the screening time^18^. Finally, the transportation of the screening device is restrictive due to its size, which makes it difficult to utilize in situations where the equipment needs to be transported between schools for SSS programmes.

This study originated from requests for an alternative screening tool to optical techniques for SSS as an additional test aside from the FBT. This new method must meet the needs of the SSS programme and the school environment and is required to be accurate, reproducible and reasonably sensitive with reliable specificity^12^. Recently, infrared (IR) thermography has been applied in numerous clinical studies. For example, studies of juvenile idiopathic arthritis^19^ and foot diabetes^20^ have both demonstrated that IR technology as a non-invasive clinical assessment tool is able to identify diseases with good reliability and sensitivity by comparing the temperature distribution difference between regions of interest. Therefore, IR thermography was specifically examined in this study as an additional detection method, as we have already performed some pilot work in a previous study in which we compared the asymmetrical paraspinal muscle activity of scoliotic patients with that of non-scoliotic subjects in terms of their daily posture with surface electromyography^21^. This type of investigation has also been performed by Chwała *et al*.^22^, as well as Farahpour *et al*. and Avikainen *et al*.^23,24^. Recent work by Zhu *et al*.^25^ on the genetic aetiology of scoliosis has also indicated that the proportion of muscle fibre type I, which is regarded as a postural muscle, is significantly lower on the concave side than on the convex side. Although asymmetrical distribution may not be the primary reason for spinal deformity, imbalance of the muscles could be a factor in scoliosis. Thus, an IR camera would be the best device for identifying differences in the paraspinal muscles through the use of temperature in scoliotic subjects.

The objective of this study was, therefore, to explore the possibility of using IR thermography to evaluate IR emissions from subjects to detect abnormalities in temperature distribution in their paraspinal muscles. It is hypothesized that IR emission should be comparatively more even in non-scoliotic subjects than in their scoliotic counterparts, as the former have a more balanced paraspinal muscle profile. For the muscle group that is tested through identical means, the temperature difference between the muscles on the left and right sides of the body is assumed to be minimal in non-scoliotic subjects. Thermal asymmetry is an indication of postural defects or a symptom of idiopathic scoliosis^26^. The advantage of using an IR camera for performing IR thermography is its ability to quantify the surface topography of subjects in terms of temperature distribution, which may be associated with muscle activity. The data obtained for analysis are objective, with a cut-off value that can be justified based on further clinical trials with blind testing. The emissivity of the IR camera can also be adjusted, which will better ensure accuracy of the measurements at different locations. The IR camera can also be easily transported and saves on the cost of hiring a professional clinician to perform the screening. Personnel with the ability and skills to operate the IR camera can perform the screening. All of these factors contribute to the feasibility of using this technique in an SSS programme. Therefore, this study supports the use of this technology in school screenings of idiopathic scoliosis and its application as an additional tool because it is non-invasive, efficient, reliable and portable.

## Results

Table 1 shows the temperature distribution in the muscles of the tested subjects, and Table 2 shows the results of the paired Student’s t-test. No significant differences were found in the muscle regions on the left and right sides of the body of the non-scoliotic subjects. However, the temperature distribution was significantly different in the scoliotic group. The results demonstrate a significant difference in the trapezius, latissimus dorsi and quadratus lumborum muscles, with a significance level of p = 0.048, 0.000 and 0.012, respectively.

**Table 1:** Temperature distribution in the muscles of the non-scoliotic and scoliotic subjects.

Group	N =	Muscle Region	Mean Temperature (°C)	S.D.
Non-Scoliotic	14	Left Trapezius	32.87	0.821
		Right Trapezius	32.92	0.784
		Left Latissimus Dorsi	32.38	0.930
		Right Latissimus Dorsi	32.46	0.941
		Left Quadratus Lumborum	31.54	0.823
		Right Quadratus Lumborum	31.60	0.821
Scoliotic	17	Left Trapezius	33.64	1.452
		Right Trapezius	33.72	1.436
		Left Latissimus Dorsi	33.00	1.46
		Right Latissimus Dorsi	33.28	1.421
		Left Quadratus Lumborum	32.25	1.645
		Right Quadratus Lumborum	32.55	1.645

**Table 2:** Results of paired Student’s t-tests for non-scoliotic and scoliotic subjects.

Group	N =	Pair	Muscle Region	Mean Difference (°C)	S.D.	Statistical significance p < 0.05
Non-Scoliotic	14	Pair 1	Left Trapezius Right Trapezius	−0.053	0.148	
		Pair 2	Left Latissimus Dorsi Right Latissimus Dorsi	−0.079	0.156	
		Pair 3	Left Quadratus Lumborum	−0.060	0.134	
			Right Quadratus Lumborum			
Scoliotic	17	Pair 1	Left Trapezius Right Trapezius	−0.077	0.149	0.048
		Pair 2	Left Latissimus Dorsi Right Latissimus Dorsi	−0.275	0.203	0.000
		Pair 3	Left Quadratus Lumborum Right Quadratus Lumborum	−0.300	0.436	0.012

## Discussion

The paired Student’s t-test results are further discussed for the two separate groups of subjects in accordance with the conducted statistical analysis. The hypothesis is that the IR emission of the same muscle region on the left and right sides of the body is similar to that in the non-scoliotic group. In contrast, the average temperature of those muscle region in the scoliotic group may be significantly different.

It was found that there was no significant difference in the temperature of the paraspinal muscles in any of the tested regions in the non-scoliotic group, while the subjects in the scoliotic group showed a significant difference in temperature in the trapezius, latissimus dorsi and quadratus lumborum muscles; the significance level was p = 0.048, 0.000 and 0.012, respectively. This result can be explained as per the work of Cooke *et al*.^26^, who found that the convex side of the observed area has a higher IR emission and therefore a higher surface temperature. The concave side has a lower IR emission; therefore, a lower surface temperature was recorded. The difference between the convex and concave sides may be the reason for the significant differences found in the tested regions. This finding could also be associated with the abovementioned findings by Zhu *et al*.^25^. The muscle fibre type I content of scoliotic individuals is significantly lower on the concave side of the curved spine than on the convex side, and this type of asymmetrical muscle fibre content leads to muscle activity imbalance along the paraspinal muscle. The relatively weaker side of the muscle could lead to decreased blood flow under controlled conditions, resulting in a lower temperature distribution than that in the relatively strong side^27^. The temperature distributions are presented in Table 3.

**Table 3:** Temperature difference between tested regions in each participant.

Subject No.	Group	ATR (°)		Ultrasound Angle (°)		IR Temperature Difference (Left - Right Side) (°C)		
		Thoracic	Lumbar	Thoracic	Lumbar	Trapezius	Latissimus Dorsi	Quadratus Lumborum
1	Non-Scoliotic	R2	R1	NA		0.03	0.02	−0.1
2		L2	0			−0.29	−0.17	−0.12
3		R3	R3			−0.17	−0.12	−0.03
4		L1	R2			−0.04	−0.03	−**0**.**34**
5		R3	R3			−0.15	−0.25	−0.14
6		R2	R4			−0.2	0.27	−0.1
7		R1	R1			0.09	0.16	0.22
8		L2	0			−0.25	−0.26	0.14
9		R3	L2			0.17	−0.05	0.03
10		0	L1			0.12	−0.04	−0.15
11		R1	0			0.05	−0.04	−0.1
12		L1	0			0.06	−0.23	−0.1
13		R2	R3			0.01	−0.24	−0.01
14		L2	L2			−0.16	−0.12	−0.03
15	Scoliotic	R4	R2	10	N/A	−0.15	−0.23	−0.1
16		L3	L7	10.8	14.6	0.06	0.05	−**0**.**48**
17		R5	R3	17.4	N/A	−0.13	−**0**.**52**	0.31
18		R7	R5	N/A	14.2	−0.12	−0.28	−**0**.**32**
19		R3	L5	N/A	21.6	−0.11	0.2	−**0**.**92**
20		R10	R3	15.2	N/A	−0.03	−0.25	0.19
21		R7	R3	N/A	13	−0.12	−**0**.**31**	−**0**.**32**
22		R5	0	13.1	20.6	−0.17	−0.2	−**0**.**5**
23		L3	L3	12.2	N/A	−0.16	−**0**.**33**	−0.2
24		L5	R3	27.6	15.4	−0.07	−0.25	0.17
25		R1	R4.5	N/A	23.8	−0.13	−**0**.**55**	−**0**.**44**
26		L3.5	L5	13	N/A	−**0**.**49**	−**0**.**49**	0.36
27		R5	R4	11.9	N/A	0.15	−0.03	0
28		R4	0	N/A	23.1	0.07	−**0**.**46**	−**0**.**83**
29		L4	R4	23.4	28.4	0.17	−0.26	−0.15
30		0	L6	16.2	18	−0.03	−**0**.**49**	−**0**.**74**
31		R5.5	R5.5	N/A	10.9	−0.04	−0.27	−**1**.**12**

^a^Bold numbers indicate that the temperature difference is more than 0.3 °C.

Based on this result, if the difference in the value of the cut-off temperature is set at 0.3 °C, the IR camera and analysis software are able to detect 12 out of the 17 cases of scoliosis. As the IR camera is sensitive enough to detect temperature differences up to 0.07 °C, the cut-off value can also be adjusted to a very precise level of differentiation.

The cut-off value can also help clinicians determine the surface topography of a patient and the need for further examination. It is also important to consider that different parameters and varying skill levels of the operator may affect the test-retest reliability. Therefore, two sets of data were acquired from the same group of subjects, and the interclass correlations (ICCs) (1,1) with 95% confidence intervals were computed for each region of interest. The results (Table 4) showed that there is reliable repeatability of the values (ICC values > 0.9).

**Table 4:** Test-retest reliability of six regions of interest.

	Left Trapezius	Right Trapezius	Left Latissimus Dorsi	Right Latissimus Dorsi	Left Quadratus Lumborum	Right Quadratus Lumborum
ICC (1,1)	0.94	0.94	0.93	0.93	0.92	0.91
CI of ICC	[0.88–0.97]	[0.87–0.97]	[0.85–0.97]	[0.85–0.96]	[0.83–0.96]	[0.82–0.96]

**Figure 1:**
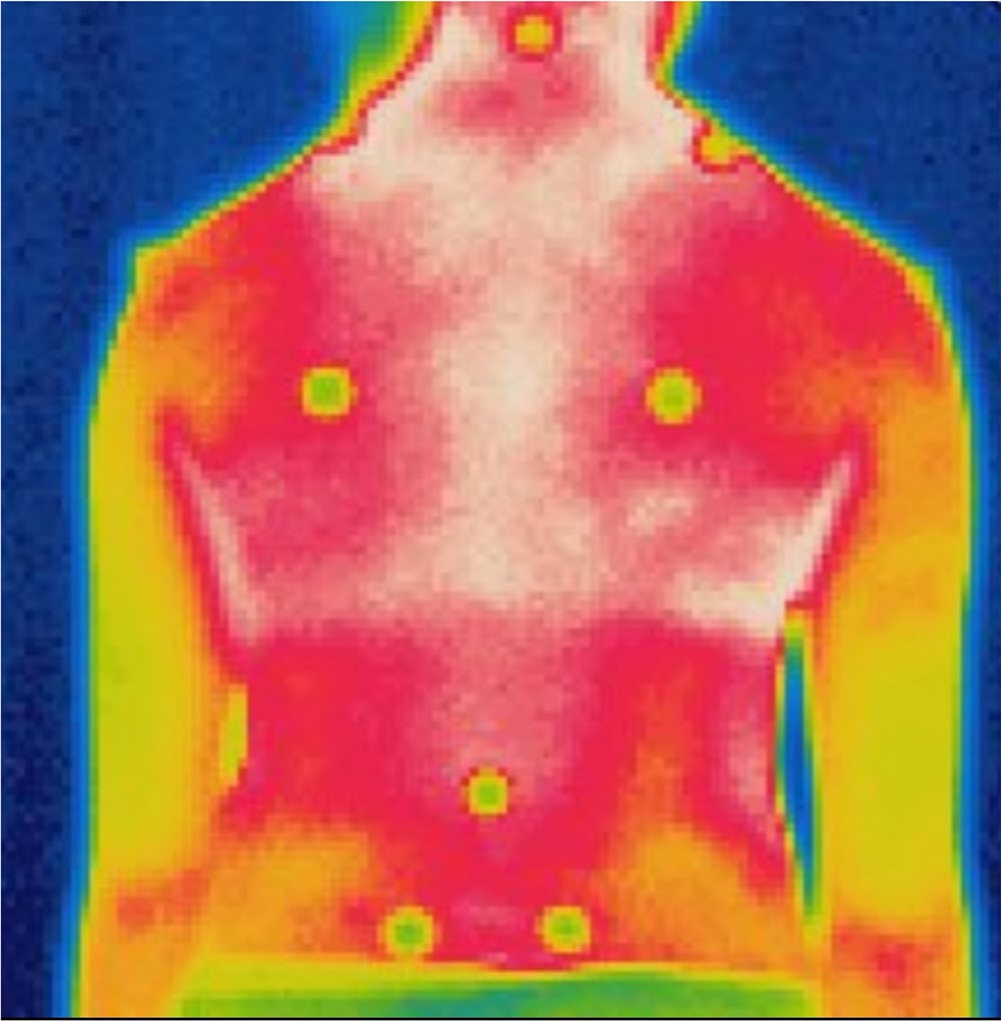
IR image of a non-scoliotic subject - symmetrical temperature distribution along the paraspinal muscles

**Figure 2:**
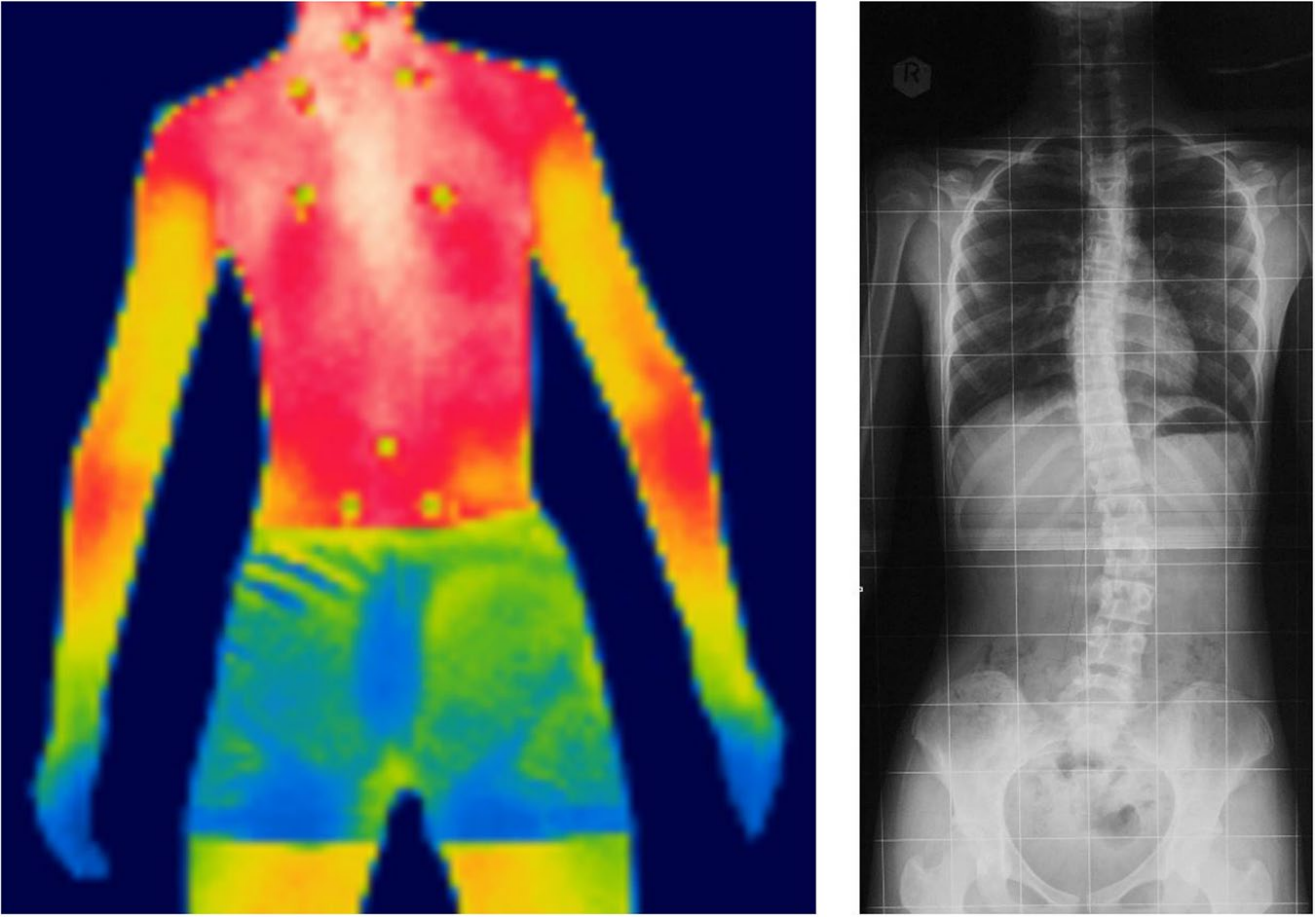
Comparison of IR images of a scoliotic subject with X-rays - S-curve of 23.6 degrees at L3 and 25.5 degrees at T9.

On the IR images, the differences in the temperature distribution profiles between the non-scoliotic and scoliotic subjects were visually apparent. Figure 1 shows an IR image obtained from a non-scoliotic subject (Subject Code: 7) that is an example of a symmetric temperature distribution along the paraspinal muscles. Her ATR at both the thoracic and lumbar regions was 1 degree towards the right side. For comparison purposes, a scoliotic subject with a curve of 23.4 degrees in the thoracic region and 28.4 degrees in the lumbar region (both obtained using the Scolioscan) was invited to undergo a radiography exam (Subject 29; see Fig. 2). The results of the exam indicated that she had an S-curve of 23.6 degrees in the lumbar region at L3 and a curve of 25.5 degrees in the thoracic region at T9. The IR image also showed an asymmetrical pattern along the paraspinal muscles. This finding correlates with the result of our previous or other related studies, in which we found that scoliotic patients have imbalanced paraspinal muscle activity^21–24^. Whether the activity of the asymmetrical paraspinal muscles is a primary or secondary reason for spinal deformity remains unknown^25^. However, the results of this study and the IR imaging also show similarities with the X-ray images of Subject 29. Therefore, it is worthwhile to further explore the possibility of using IR thermography as a scoliosis screening tool by clinicians.

In this study, the possibility of using IR thermography as an alternative SSS technique to detect potential scoliosis cases among adolescents was examined. IR thermography requires approximately 5 minutes for each patient, including the time required to locate nine body landmarks and take three IR images. The preparation time is similar to that of existing screening tools^7^. IR thermography can be implemented by one technician with basic equipment operation knowledge. In terms of feasibility, IR thermography provides similar outcomes to those of the FBT and Moiré topography, which identify the spinal structure based on the surface profile of the subject with varying degrees of accuracy^28^. Based on the results of this study, IR thermography can reveal significant differences between the paraspinal muscles on the left and right sides of the body in scoliotic subjects, including in the thoracolumbar region where scoliotic conditions are commonly found^22^. The equipment also provides excellent repeatability, with an ICC level over 0.9 based on the two sets of data collected from the same group of subjects.

IR thermography also provides another advantage in terms of mobility. IR cameras are compact and easy to set up. The IR camera used in this experiment is lightweight and can be mounted onto a standard camera tripod. All of the images are stored onto a memory card. The mobility of IR cameras means that the location of screenings is not limited to one place, enabling scoliosis screening to be performed in different schools.

The proposed method in this article establishes some of the groundwork for using IR thermography in detecting the signs of scoliosis. The advantage of using an IR camera is its ability to quantify the surface topography of the participants in terms of temperature distribution, which may be associated with muscle activity. The data obtained for analysis are objective in nature, with a cut-off value that can be justified based on further clinical trials with blind testing.

The emissivity level of the IR camera can also be adjusted, which will ensure better accuracy of the measurements at different locations. The IR camera can also be easily transported and saves on the cost of hiring a professional clinician to perform the screening, as personnel with the ability and skills to operate the IR camera can perform the screening. In addition, there are examples of using automated algorithms to analyse the collected IR data, which could minimize intra-observer error^20,29^ and demonstrate the potential of using IR in clinical assessments. All of these factors contribute to the feasibility of using this technique in an SSS programme.

However, this study has some limitations, including the small number of subjects recruited, rendering the study findings non-generalizable. It is recommended that the number of both non-scoliotic and scoliotic subjects be increased. In this way, the false-negative and false-positive rates with the use of this technique can be examined through a ‘blind’ interpretation of the IR images. A larger sample can also help to define the indices that would differentiate between potential scoliotic patients and non-scoliotic subjects. The optimal cut-off value of the temperature can be further refined with a larger group. Moreover, the acclimatization time is an area that warrants further exploration, as in this study, the acclimatization time was recommended to be no less than 20 minutes in a conditioned room. This parameter is adopted from research work by Abate *et al*.^30^, who used IR thermography to capture postural adjustments in leg length difference. Roy *et al*.^31^, who used an acclimatization time of 8 to 12 minutes, found that this range is the minimum requirement for a stable recording. Therefore, further studies are required to determine the exact duration for SSS. Furthermore, the gold standard for diagnosing scoliosis is radiography, rather than Scolioscan, which is a tool used to provide the spinal deformity angle of the participants, despite the demonstration by the development team of Scolioscan showing that the scanner has good reliability and close correlations with radiography^32–34^.

Nevertheless, this study still provides evidence of the feasibility of incorporating IR thermography into school screening for scoliosis.

## Methods

### Subjects

A pre-screening programme was conducted in two local schools to collect demographic data and the FBT results of students between the ages of 10 and 13 years. The subjects were asked to perform the FBT, and a clinician used a scoliometer to measure the rib hump, which is correlated with spinal rotation and rib deviation. The ATR was recorded in the thoracic and lumbar regions of the participants. Subjects with an ATR over 3 degrees were invited to participate in ultrasonic imaging using Scolioscan (Telefield Medical Imaging Limited, Hong Kong)^32–34^ to further investigate their spinal status. The Scolioscan uses ultrasound to create a lateral spinal image. The spinal deformity angle and rotation can be measured through this tool without exposing the participants to any radiation. In total, 185 female students were screened, and 26 were found to have an ATR over 3 degrees (14.1%). Seventeen individuals with a spinal curve that exceeded 10 degrees were recruited. Fourteen non-scoliotic female adolescent volunteers were also recruited as the control group. The characteristics of the subjects are provided in Table 5. The study was conducted in accordance with the Helsinki Declaration of the World Medical Association Assembly, and the research protocol was approved by the Human Subjects Ethics Sub-committee (HSESC) of the Hong Kong Polytechnic University. All the subjects participated voluntarily and were required to sign informed consent forms prior to the study, in accordance with institutional guidelines.

**Table 5:** Demographic data of recruited subjects.

Group	N =	Upper extremity dominant side	Height (cm) (S.D.)	Weight (kg) (S.D)	BMI (S.D)	ATR (°) (S.D)		Ultrasound Angle (°) (S.D)	
						Thoracic	Lumbar	Thoracic	Lumbar
Non-Scoliotic	14	Right	154.7 (6.1)	47.66 (6.90)	19.86 (2.26)	1.79 (0.86)	1.57 (1.29)	N/A	
Scoliotic	17		154.5 (4.75)	44.51 (5.94)	18.65 (2.40)	4.41 (2.24)	3.71 (1.85)	15.53 (5.23)	18.51 (5.16)

### IR camera and experimental procedures

**Figure 3:**
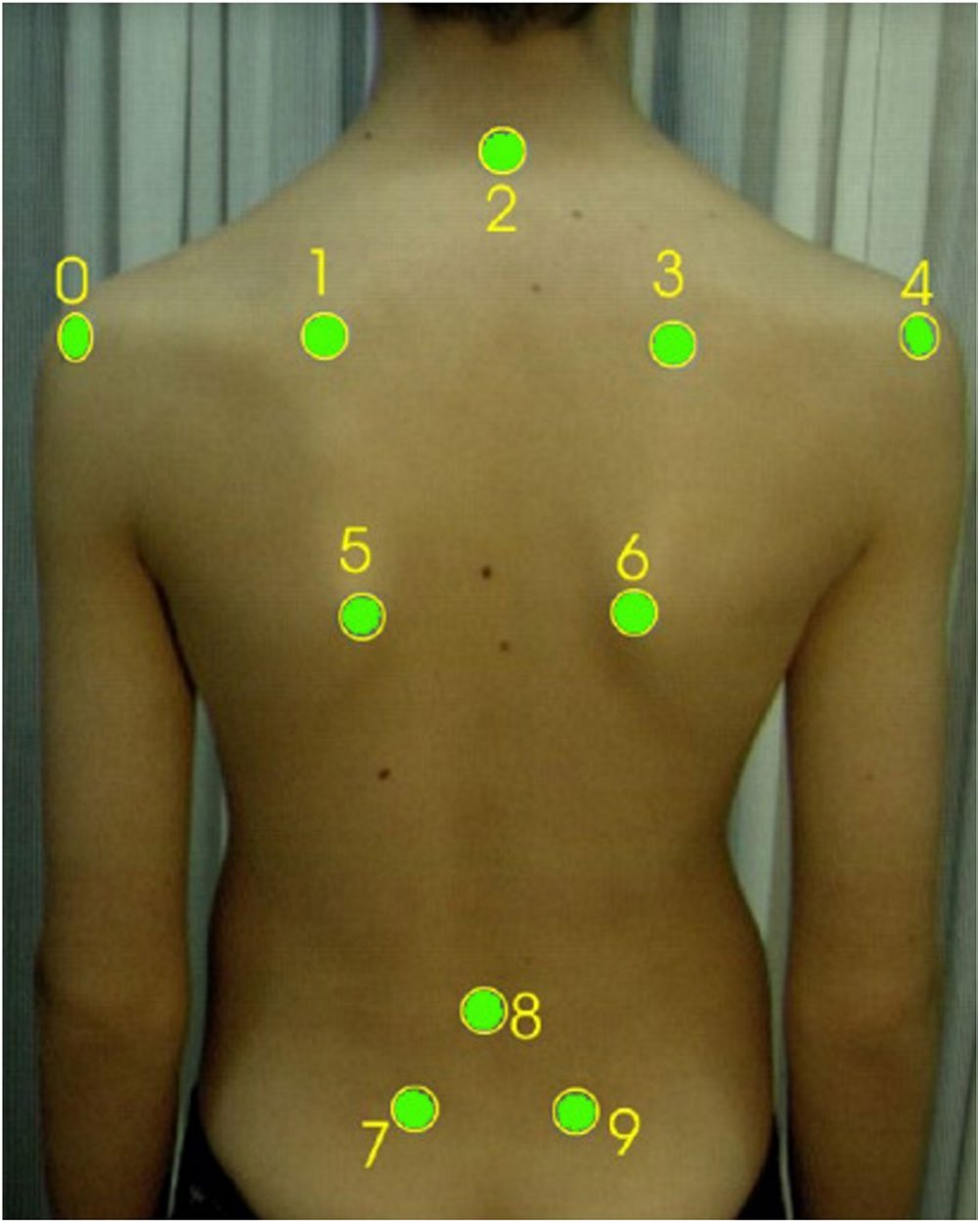
Location of body landmarks per SOSORT (Source: Patias *et al*.^18^).

The experiment was conducted in a conditioned room at 20 °C ± 2 °C and a relative humidity of 55%^30^. The subjects were asked to undress, and a physiotherapist pinpointed the recommended landmarks on their body per the Society on Scoliosis Orthopaedic and Rehabilitation Treatment (SOSORT) guidelines^18^ (Fig. 3). The body landmarks acted as a guide with which to identify the target muscle regions. Acclimatization was achieved by allowing the subjects to rest in a conditioned environment for 20 minutes. The participants were instructed to avoid beverages that contain caffeine 4 hours before the experiment and to avoid exercise before the recording session^31^.

IR imaging was performed with an FLIR E33 camera (FOL-18 lens; 10,800 pixels). The thermal sensitivity was 0.07 °C. The camera was placed onto a tripod, with a distance between the camera and subjects of 1.5 m. The IR camera emissivity level was set to 0.98. IR imaging was used to capture the entire temperature distribution in the paraspinal muscles.

### Data analysis

**Figure 4:**
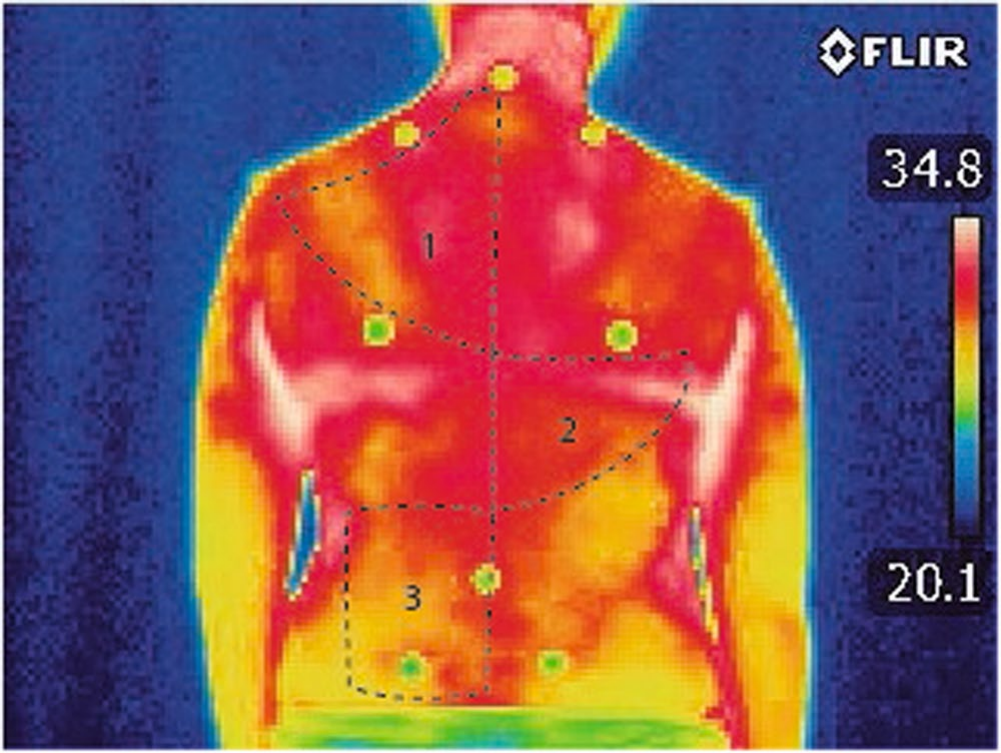
Regions of interest computed with Thermacam Researcher Professional 2.9 software. 1 is the trapezius, 2 is the latissimus dorsi, and 3 is the quadratus lumborum. Average and standard deviations of regions obtained bilaterally.

The IR images were analysed using the Thermacam Researcher Professional 2.9 Software (FLIR). The regions of interest, which included the left and right trapezius, latissimus dorsi and quadratus lumborum (Fig. 4) muscles^30^, were located with the aid of the body landmarks. The software computed the average and standard deviations of the temperature distribution within each region, as indicated in Fig. 2 with black dotted lines. The data were obtained bilaterally for each region of interest.

### Statistical analysis

The data were inputted into the SPSS 19 software for Windows to compare the distribution of the bilateral muscle temperature between the two groups of subjects. Paired Student’s t-tests were used to compare the bilateral temperature differences. The statistical analysis had a significance level of p < 0.05.
